# Deletion of mouse MsrA results in HBO-induced cataract: MsrA repairs mitochondrial cytochrome c

**Published:** 2009-05-15

**Authors:** LA Brennan, W Lee, T Cowell, F Giblin, M Kantorow

**Affiliations:** 1 Biomedical Sciences Department, Charles E. Schmidt College of Biomedical Science, Florida Atlantic University, Boca Raton, FL; 2 Eye Research Institute, Oakland University, Rochester, MI

## Abstract

**Purpose:**

Considerable evidence indicates a role for methionine sulfoxide reductase A (MsrA) in lens cell resistance to oxidative stress through its maintenance of mitochondrial function. Correspondingly, increased protein methionine sulfoxide (PMSO) is associated with lens aging and human cataract formation, suggesting that loss of MsrA activity is associated with this disease. Here we tested the hypothesis that loss of MsrA protein repair is associated with cataract formation. To test this hypothesis we examined the effect of MsrA deletion on lens opacity in mice treated with hyperbaric oxygen, identified lens mitochondrial proteins oxidized upon deletion of MsrA and determined the ability of MsrA to repair the identified proteins.

**Methods:**

Wild-type and MsrA knockout mice were treated or not treated with 100 treatments of hyperbaric oxygen (HBO) over an 8 month period and lenses were examined by in vivo light scattering measurements documented by slit-lamp imaging. Co-immunoprecipitation of MsrA was conducted against five specific protein representatives of the five complexes of the electron transport chain in addition to cytochrome c (cyt c). Cyt c in lens protein from the knockout and wild-type lenses was subjected to cyanogen bromide (CNBr) cleavage to identify oxidized methionines. Methionine-specific CNBr cleavage was used to differentiate oxidized and un-oxidized methionines in cyt c in vitro and the ability of MsrA to restore the activity of oxidized cyt c was evaluated. Mass spectrometry analysis of cyt c was used to confirm oxidation and repair by MsrA in vitro.

**Results:**

HBO treatment of MsrA knockout mice led to increased light scattering in the lens relative to wild-type mice. MsrA interacted with four of the five complexes of the mitochondrial electron transport chain as well as with cyt c. Cyt c was found to be aggregated and degraded in the knockout lenses consistent with its oxidation. In vitro analysis of oxidized cyt c revealed the presence of two oxidized methionines (met 65 and met 80) that were repairable by MsrA. Repair of the oxidized methionines in cyt c restored the activity of cytochrome c oxidase and reduced cytochrome c peroxidase activity.

**Conclusions:**

These results establish that MsrA deletion causes increased light scattering in mice exposed to HBO and they identify cyt c as oxidized in the knockout lenses. They also establish that MsrA can restore the in vitro activity of cyt c through its repair of PMSO. These results support the hypothesis that MsrA is important for the maintenance of lens transparency and provide evidence that repair of mitochondrial cyt c by MsrA could play an important role in defense of the lens against cataract formation.

## Introduction

Significant evidence points to a role for methionine sulfoxide reductases (Msrs) in diseases of aging including age-related cataract of the eye lens. Msrs are a family of thioredoxin dependent oxidoreductases that reduce the oxidized form of protein methionine, protein methionine sulfoxide (PMSO), back to its reduced form, methionine. Two classes of Msrs are known; MsrA and MsrB which act on the S- and R- epimers of PMSO, respectively. The PMSO content increases with age in a number of tissues and aging models [1] including the lens [2] and it has been shown that increased levels of PMSO are associated with age-related cataract [2,3] where the PMSO content of the cataractous lens is as high as 70% of total soluble lens proteins.

Levels of MsrA and MsrA activity decrease with age in rat tissue [4] and in the brains of Alzheimer’s patients [5]. MsrA is also known to modulate lifespan in animals, for example, MsrA knockout mice have been reported to have a 40% reduction in lifespan relative to wild-type mice [6] and exhibited increased sensitivity to oxidative stress (100% oxygen) with increased levels of oxidized proteins. In addition, the MsrA knockout mice developed an atypical (tip-toe) walking pattern after 6 months of age indicative of neuronal damage. Over-expression of MsrA in *Drosophila melanogaster* was shown to increase lifespan by up to 70% [7] and causes increased oxidative stress resistance in WI-38 SV40 fibroblasts [8], yeast and human T cells [9], and human lens epithelial cells [10]. Silencing of the MsrA gene using siRNA increased sensitivity of human lens epithelial cells to H_2_O_2_-induced oxidative stress [10]. In addition, this loss of MsrA resulted in loss of mitochondrial membrane potential, increased mitochondrial ROS production, and decreased lens cell viability [11] all of which occurred without exogenously added oxidative stress, leading to the hypothesis that lens cells require MsrA for both normal mitochondrial maintenance and viability. These data, in conjunction with data showing increased PMSO upon human lens aging and cataract formation, suggest that MsrA activity is important for lens maintenance and defense against oxidative stress through its repair of oxidized lens mitochondrial proteins. Identification of those lens mitochondrial proteins repaired by MsrA could provide insight into the requirement for MsrA in maintenance of the lens and prevention of cataract formation.

One key model for studying oxidative stress is hyperbaric oxygen (HBO) treatment. HBO-treatment has been shown in a number of studies to cause mitochondrial dysfunction, increased mitochondrial ROS production, decreased antioxidant ability and viability in lens epithelial cells, decreased thioredoxin reductase activity, and, importantly, decreased glutathione (GSH) levels, a key event in cataract formation [12-14].

Here, we used HBO-treatment to induce oxidative stress in the lenses of MsrA knockout and wild-type mice. Since mitochondrial maintenance appears to depend on the function of MsrA in lens cells [11], we examined potential mitochondrial protein-MsrA interactions and determined that cytochrome c (cyt c) was oxidized in the MsrA knockout lenses. Cyt c is a small globular heme-containing electron carrier in the electron transport chain of the mitochondria. In addition to its requirement for energy production, its release from the mitochondria to the cytosol is the initiating factor for the internal pathway of apoptosis [15]. Thus, oxidation of cyt c in lens cells could lead to loss of lens epithelial functions requiring ATP and aerobic mitochondrial function, including cationic transport and potentially epithelial cell growth and differentiation. Consistent with being a potential target for MsrA, the hexa-coordinated arrangement of heme iron in cyt c relies on methionine 80 (met 80). The heme iron moiety of cyt c is protected by this arrangement preventing binding of potentially reactive molecules such as NO, O_2_, carbon monoxide (CO), and H_2_O_2_ [16]. The met 80 of cyt c is therefore a key amino acid in the structure and function of the molecule and previous work by Chen et al. [17] demonstrated that hypochlorous acid (HOCl)-mediated oxidation of cyt c resulted in the specific oxidation of cyt c met 80 to met 80 sulfoxide and that this oxidation increased the accessibility of the molecule to H_2_O_2_ due to the inability of the met 80 sulfoxide to co-ordinate the heme iron.

In this report, we examined the lenses of MsrA knockout mice in the presence and absence of hyperbaric oxygen treatment and found increased light scattering in the knockout relative to the wild-type lenses. We identified 5 major components of the electron transport chain that interact with MsrA in lens cells as well as the electron carrier protein cyt c. Analysis of lens protein obtained from untreated wild type and knockout mouse lenses revealed an increased level of oxidized cyt c occurring in the absence of MsrA in vivo. Following HBO treatment, soluble monomeric cyt c was no longer detectable in the lenses of MsrA knockout mice. Further analysis of oxidized cyt c in vitro demonstrated that methionine oxidation of cyt c resulted in loss of activity that was repairable by treatment with purified MsrA. These results provide evidence for a novel role for MsrA in the maintenance of lens transparency in the face of oxidative insult. They also demonstrate that oxidation of cyt c occurs in the lens in vivo in the absence of MsrA and that MsrA can restore its normal function in vitro. The results also suggest that cyt c is at least one target for MsrA in lens epithelial cells whose repair by MsrA is likely important for maintaining lens mitochondrial function and preventing cyt c-mediated apoptosis. These findings shed light on the potential roles of MsrA and cyt c in the development of lens cataract and are relevant towards the understanding of MsrA action and PMSO accumulation in other age-related oxidative stress associated diseases.

## Methods

### HLE B3 cell culture

Human lens epithelial cells (HLE B3) were grown and cultured in DMEM (Invitrogen, Carlsbad, CA) supplemented with 15% FBS (Invitrogen), gentamicin (50 units/ml; Invitrogen), penicillin-streptomycin antibiotic mix (50 units/ml; Invitrogen) and fungizone (5 ul/ml; Invitrogen) at 37 °C in the presence of 5% CO_2_.

### HBO treatments

Wild type and MsrA knockout mice were treated with HBO in a pressure vessel 130 cm long and 46 cm in diameter (Amron, Escondido, CA). The vessel had a fully opening hinged door at one end and at the opposite end, a 15 cm viewport for observing animals during the treatment. The light was kept off during the treatment, and the window was covered. The mice were housed in wire cages with tops during the oxygen exposure. Plastic trays containing wet towels were placed inside the chamber to add humidity, and Sodasorb (WR Grace, Lexington, MA) was added to absorb CO_2_. After the chamber was sealed, it was flushed for 5 to 10 min with approximately 1 volume of 100% O_2_ (USP Grade Medical Gas; Praxair, Danbury, CT), which was vented outside the building. The pressure was then raised during the next 15 min to 2.75 atm absolute (ATA; 26 pounds per square inch gauge or 58 ft of sea water) of O_2_. After 1.5 h, the chamber was flushed with approximately 1 volume of fresh 100% O_2_, while the pressure was maintained. At the end of a 3.0 h holding period the pressure was released over a 15 min period to 1 ATA (0 psig), and the animals removed. The mice were treated three times per week, on alternate days at approximately the same time of day, for a total of 100 treatments in 8 months. Age-matched control animals were included with each group of O_2_-treated mice. The transparency of the lenses of control and HBO-treated mice was assessed in vivo with a slit lamp microscope (Carl Zeiss, Meditec, Thornwood, NY) after induction of full mydriasis with tropicamide (1%) and phenylephine (10%), and the results documented by photography. After the animals were killed by CO_2_ asphyxiation, the eyes were enucleated and the lenses removed by a posterior approach, and frozen immediately in dry ice.

MsrA knockout (C57 Black 6, C57BL/6) [6] (a gift from Dr. Rodney Levine, NIH, Bethesda, MD) and wild type mice (B6129SF2/J strain) were sacrificed following HBO treatment at 8 months and the eyes enucleated. Lenses were removed and frozen on liquid nitrogen. Lens proteins were isolated by homogenization in PBS, homogenates were centrifuged at 10,000x g for 30 min at 4 °C and the supernatant collected. Soluble lens protein extracted in this way was subjected to cyanogen bromide (CNBr) cleavage and cyt c analyzed by western blotting using a cyt c specific antibody (R&D systems, Minneapolis, MN). The epitope for the antibody does not incorporate either of the two methionines potentially oxidized in cyt c (65 or 80). Western blotting of oxidized purified cyt c protein without CNBr treatment shows that the antibody continues to recognize the protein even at high oxidant levels. Corresponding colloidal blue staining of the same gels shows that protein destruction occurs before loss of antibody recognition.

### Co-localization of MsrA to the mitochondria and with cytochrome c in HLE cells

HLE cells were plated onto coverslips and incubated overnight in complete media. Standard procedures were used for double immunofluoresence-simultaneous staining. Briefly, cells were fixed with 3.7% formaldehyde in PBS, blocked with 1% BSA and permeabilized with 0.25% TritonX-100 in PBS. For co-localization of MsrA to the mitochondria, first, HLE cells were stained with Mitotracker Red CMXRos (Molecular Probes, Invitrogen) for 45 min at a 1:500 dilution as indicated by the manufacturer’s protocol. Following mitotracker red staining, rabbit polyclonal anti-MsrA (Abcam, Cambridge, MA), was incubated overnight in 4 °C at a 1:100 dilution. For co-localization of MsrA and cyt c in the same HLE cells, rabbit polyclonal anti-MsrA (Abcam) was incubated simultaneously with mouse monoclonal anti-cytochrome c antibody (Mitosciences, Eugene, OR) overnight in 4 °C at 1:1,000 dilutions. Cells were washed 3X with PBS, and then incubated with Alexa Fluor 488 goat anti-mouse monoclonal secondary and Texas Red goat-anti Rabbit polyclonal secondary for one hour at room temperature at 1:2,000 dilutions. HLE cells were washed with PBS 3X, and mounted onto slides. Immunofluoresence staining was visualized using an Olympus Provis AX70 fluorescence microscope.

### Isolation of mitochondria from HLE B3 cells

Mitochondria were isolated from HLE B3 cell culture using the Isolation of Mitochondria from Cell Culture kit (Mitosciences) as per the manufacturer's instructions. Briefly, cells were frozen and thawed prior to re-suspension in isolation buffer at a concentration of 5 mg/ml and lysed by Dounce homogenization. Lysates were incubated in isolation buffer with 20 mM n-dodecyl-β-D-maltopyranoside for 30 min on ice followed by centrifugation at 20,000x g for 30 min at 4 °C. Protein and mitochondria (in 50 mM Tris-HCl [pH 7.4], 150 mM NaCl, 2 mM EGTA, 2 mM EDTA, 0.2% Triton X-100 and 0.3% NP-40) concentrations were determined by Bradford protein assay.

### Immunoprecipitations

Antibodies specific to individual proteins were conjugated to protein-G agarose beads (Complexes I-V; Mitosciences) or NHS activated beads (MsrA; Covance Research Products, Princeton, NJ). Complex I-V antibodies were directed against specific subunits of each protein as follows: complex I – NDUFS4 (18 kDa), complex 2 – 70 kDa subunit, complex III – core I subunit (48 kDa), complex IV – cytochrome c oxidase subunit I (57 kDa), and complex V – ATP synthase subunit beta (56 kDa). MsrA antibody used for conjugation was purchased from AbCam. HLE B3 mitochondrial protein (500 µg) or 500 µg of bovine liver tissue was incubated overnight at 4 °C in a test tube rotator at a concentration of 1 mg total protein per 100 µg antibody. Beads were collected by centrifugation at 1,000x g for 1 min and washed 4X with 1X PBS 1 mM n-dodecyl-β-D-maltopyranoside (nDMP). Protein was eluted by incubating for 10 min in 0.2 M Glycine 1 mM n-DMP buffer pH 2.5 with frequent agitation. Elution was repeated 3 times prior to washing the beads and storing them in PBS 0.05% sodium azide for future use. Eluted protein analyzed by SDS-PAGE and western blotting.

For cyt c immunoprecipitation, 500 µg of HLE B3 mitochondrial protein was incubated with 20 µg anti-cytochrome c antibody (Abcam) for 1 h at 4 °C while rotating. The mixture was then added to 25 µl packed Protein A-agarose beads (100 µl bead matrix; Sigma, St, Louis, MO) pre-equilibrated with RIPA buffer and mixed by rotation at 4 °C overnight. The beads were then collected by centrifugation at 8,000 rpm for 30 s in a microcentrifuge. The supernatant was saved for further analysis. The beads were washed 3 times in 1 ml 1X PBS 1 mM n-DMP (Sigma-Aldrich). Protein was eluted by boiling for 10 min in 40 µl 2X SDS loading buffer. Eluted protein was analyzed by SDS-PAGE and western blotting.

### Identification of methionine residues of oxidized cytochrome c

CNBr hydrolyzes peptide bonds at the COOH-terminus of methionine residues; it does not recognize methionine sulfoxide, the oxidized form of methionine, leading to a lack of cleavage. Thus CNBr cleavage is a sensitive detection method to identify methionine sulfoxide [18]. Purified mouse MsrA was obtained as a gift from Dr. Rodney Levine. Horse heart cyt c protein (0.2 mM; Sigma-Aldrich) was incubated with hypochlorous acid (HOCl) with increasing molar ratios (2:1, 4:1, and 6:1) at room temperature for 15 min. For repair of oxidized methionines, DTT (15 mM) was used in the repair buffer as the reducing system for the MsrA enzyme. Oxidized cyt c (291 µM) was repaired using 1.9 µM MsrA for 2 h at 37 °C. Samples were dried on a vacuum centrifuge and diluted in 70% formic acid to 10-20 mg/ml. CNBr (Sigma-Aldrich) was added in a 2:1 w/w ratio and the reaction mix incubated at room temperature for 24 h in the dark. The reaction was terminated by addition of 5 volumes ddH_2_O and 5 volumes 1 M ammonium bicarbonate. Samples were then concentrated using an Amicon stirred ultrafiltration cell (Amicon, Millipore, Bedford, MA). Each cyt c sample (5 µg) was run on a 16% Tricine-SDS-PAGE gel at 90 mV. The gels were stained with Colloidal blue for 4 h and de-stained in ddH_2_O overnight.

### Enzyme assays

Cytochrome c oxidase activity was measured using a commercially available kit (Sigma-Aldrich) and following the manufacturer’s instructions. The absorption of cyt c changes with its oxidation state, this property is the basis of the cytochrome c oxidase assay. Cyt c (horse heart; Sigma-Aldrich) was treated with HOCl and subsequently MsrA/DTT as follows: cyt c was incubated with HOCl in a 4:1 ratio (0.8 mM HOCl:0.2 mM cyt c) for 15 min at room temperature. After incubation, the excess HOCl was removed from the oxidized cyt c using ultrafiltration on an Amicon cell. Oxidized cyt c (291 µM) was incubated with MsrA (1.9 µM) for 2 h at 37 °C in the presence of DTT (15 mM). DTT alone in the repair buffer had no effect on oxidase activity using oxidized cyt c.

The enzymatic peroxidase activity was measured by ABTS oxidation. The effect of oxidation on the peroxidatic activity of cyt c was assayed by adding 0.6 µM cyt c to 1 ml of the mixture containing 1.3 mM ABTS, 12 mM H_2_O_2_, and 100 µM diethylenetriaminepentaacetic acid in 100 mM phosphate buffer (pH 7.4) at 23 °C, followed by measuring the increase in absorbance at 420 nm over 60 s [19]. Cyt c samples were prepared exactly as described for the oxidase assay. Some effect of decreasing peroxidase activity was seen with DTT alone in the repair buffer; this is not shown but taken into account when presenting the results of oxidized cyt c repaired by MsrA. Mass spectrometry used to confirm oxidation of cyt c was carried out by Dr. Rodney Levine at the NIH.

### SDS-PAGE, Tris-Tricine gel electrophoresis, and western blotting

Unless specified, all electrophoresis reagents and apparatus were purchased from Bio-Rad (Richmond, CA). Protein samples were mixed with 2X sample buffer at a 1:1 volume ratio and heated at 100 °C for 5 min. The samples were separated by electrophoresis on either 15% (immunoprecipitations) or 17% (CNBr samples) Tricine–sodium dodecyl sulfate (SDS) gels at room temperature for 1.5 h at 120 volts. Tricine-SDS-PAGE gels were used for better separation of low molecular weight CNBr products of cyt c. For Tricine-SDS-PAGE gel electrophoresis the method of Schagger [20] was used.

Where appropriate, gels were transferred onto nitrocellulose membranes (Amersham-Pharmacia, Piscataway, NJ) using a Mini Trans-Blot electrophoresis transfer cell apparatus (Bio–Rad). The transfers were performed at 100 volts in 25 mM Tris, 192 mM glycine, 20% methanol at room temperature for 2 h. The membrane was equilibrated in Tris-buffered saline (TBS), pH 7.4, 0.05% Tween-20 for 15 min then blocked in TBS (pH 7.4), 5% Carnation nonfat milk, and 0.05% Tween-20, for 1 h. The membrane was incubated with the appropriate antibody overnight or for anti MsrA for 2 h, followed by incubation for 1 h with the appropriate secondary antibodies (Sigma-Aldrich). In between incubations, three washes of 15 min and two of 5 min were performed with TBS, 0.05% Tween-20. The blot was washed and visualized using ECL western blotting reagents (Amersham-Pharmacia) as specified by the manufacturer. The following antibody concentrations were used where appropriate: primary anti-MsrA 1:2,500, primary anti-cytochrome c 1:10,000 primary anti-NDUFS4 (Complex I) 1:5,000, primary anti-Complex II 70 kDa subunit 1:10,000, primary anti-core I subunit (Complex III) 1:5,000, primary anti-subunit I complex IV 1:1,000, primary anti-subunit beta Complex V 1:5,000, secondary anti-mouse (cytochrome c) or anti-rabbit (all others) 1:2,000. Gels were also stained with Colloidal blue for 4 h and de-stained overnight in ddH_2_O.

## Results

### Deletion of MsrA leads to increased light scattering in mouse lenses treated with hyperbaric oxygen

**Figure 1:**
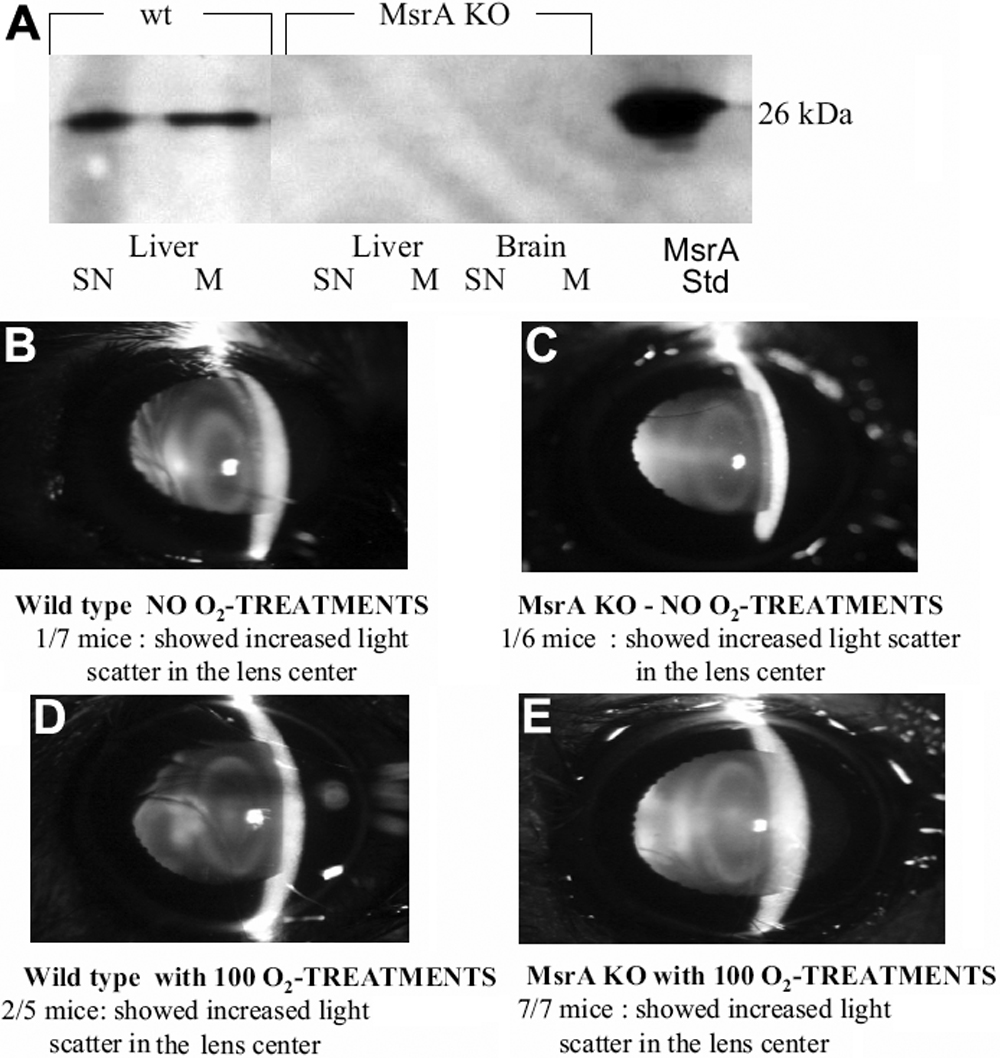
Representative slit lamp images of the lenses of MsrA knockout mice and wild-type mice exposed to hyperbaric oxygen. **A**: Western blot of liver tissue supernatant (SN) and mitochondrial protein (M) isolated from wild type mice and liver and brain tissue supernatant (SN) and mitochondrial protein (M) isolated from MsrA knockout mice using an MsrA specific antibody. **B**-**E**. Slit lamp microscopy of mouse lenses. Indicated are numbers of mice photographed, number of HBO treatments and numbers of mice exhibiting light scattering after 8 months of treatment.

Wild-type and MsrA-knockout mice were phenotyped to confirm lack of MsrA protein expression by western analysis using an MsrA-specific antibody. As shown in Figure 1A, MsrA was detected in wild-type liver supernatant (SN) and isolated mitochondrial fraction (M) but not in corresponding fractions of brain or liver isolated from the MsrA knockout mice. Knockout and wild-type mice were treated with 2.5 ATM of hyperbaric oxygen three times per week, on alternate days at approximately the same time of day, for a total of 100 treatments over a 8 month period. The transparency of the lenses of control and HBO-treated mice was assessed by in vivo slit lamp microscopy and the results documented by photography. Figures 1B-E show representative lenses from wild-type and MsrA-knockout mice treated or not with HBO. Light scattering was not significantly increased in the wild-type lenses with (Figure 1D) or without (Figure 1B) HBO exposure. The knockout lenses not exposed to HBO-induced oxidative stress (Figure 1C) did not show increased light scatter compared to wild-type mice. By contrast, increased light scattering was detected in the MsrA knockout mice exposed to HBO-induced oxidative stress (Figure 1E) suggesting an increased sensitivity of MsrA-knockout mouse lenses to HBO-induced oxidative stress.

### MsrA co-immunoprecipitates with multiple lens mitochondrial complex proteins including cytochrome c in vitro.

The presence of light scattering in the MsrA-knockout mice treated with HBO indicates that one or more lens proteins are aggregated by oxidation occurring in the absence of MsrA activity. Previous studies demonstrated that MsrA is required for lens epithelial cell mitochondrial function [11] and lens cell resistance to oxidative stress damage [10], suggesting MsrA may act on one or more mitochondrial targets.

**Figure 2:**
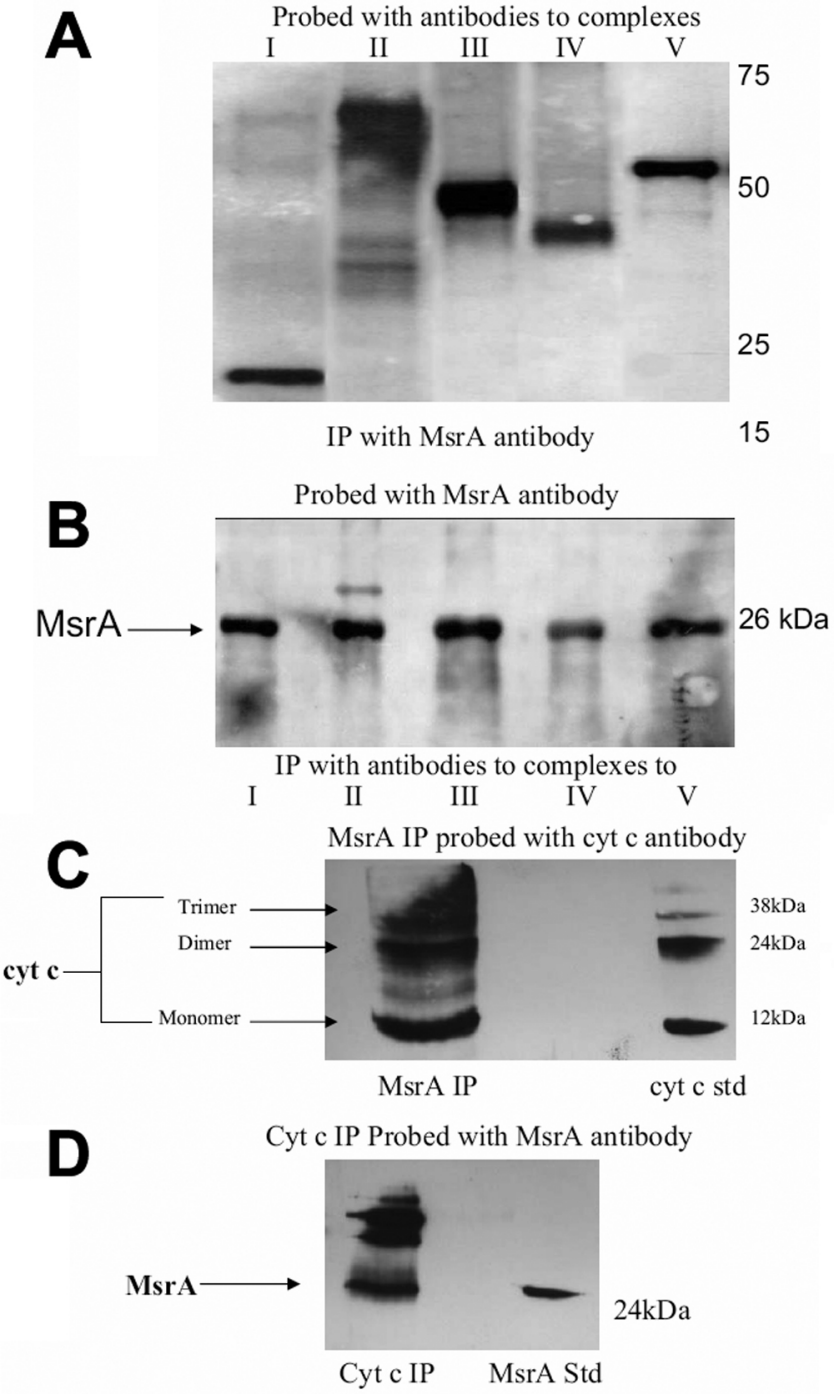
MsrA interacts with the components of the electron transport chain in human lens epithelial cells. **A**: SDS-PAGE and immunoblotting of HLE mitochondrial protein immunoprecipitated using an antibody to MsrA was probed with antibodies to specific subunits of complexes I-V (CI=NDUFS4 [18 kDa], CII=70 kDa subunit, CIII=core 1 subunit [48 kDa], CIV=cytochrome c oxidase subunit I [57 kDa], CV=ATP synthase subunit beta [56 kDa]). **B**: SDS-PAGE and immunoblotting of bovine liver tissue supernatant immunoprecipitated using antibodies to specific subunits of complexes I-V (listed above) was probed with an antibody to MsrA. **C**: SDS-PAGE and immunoblotting of HLE mitochondrial extracts immunoprecipitated using an MsrA-specific antibody and probed with a cyt c-specific (12 kDa) antibody. **D**: SDS-PAGE and immunoblotting of HLE mitochondrial extract immunoprecipitated using a cyt c-specific antibody and probed with an MsrA-specific antibody.

To determine potential mitochondrial targets of MsrA in lens cells, co-immune precipitations on HLE B3 mitochondrial extracts were carried out using MsrA antibody, and probed with specific antibodies against proteins comprising the five mitochondrial respiratory complexes (complex I – NDUFS4 (18 kDa), complex 2 – 70 kDa subunit, complex III – core I subunit (48 kDa), complex IV – cytochrome c oxidase subunit I (57 kDa), and complex V – ATP synthase subunit beta (56 kDa) and the electron carrier cyt c. The data shows that complexes I, II, III and V were co-immunoprecipitated with the MsrA antibody (Figure 2A). The complex IV protein was smaller than predicted and therefore cannot be confirmed. As a control Figure 2B shows co-immunoprecipitation of bovine liver tissue with antibodies to the five complexes and probed with MsrA. This blot shows that all five complexes co-immunoprecipitate with MsrA in bovine liver tissue. MsrA also co-immunoprecipitated with cyt c (Figure 2C,D) in the mitochondria of human lens epithelial cells. Interestingly, non-reducible dimer and trimer forms of cyt c were detected in the MsrA immunoprecipitate. Multiple protein subunits compose each of the respiratory complexes all of which are candidates for MsrA action.

In addition to the complexes, MsrA interacted with cyt c. Cyt c acts as both an electron carrier and a regulator of apoptosis [21], and since energy production [22] and apoptosis [23-25] are believed to be involved in cataract formation, we chose to study cyt c as a potential target for MsrA action in the lens.

### MsrA and cytochrome c co-localize in lens cells in vivo

**Figure 3:**
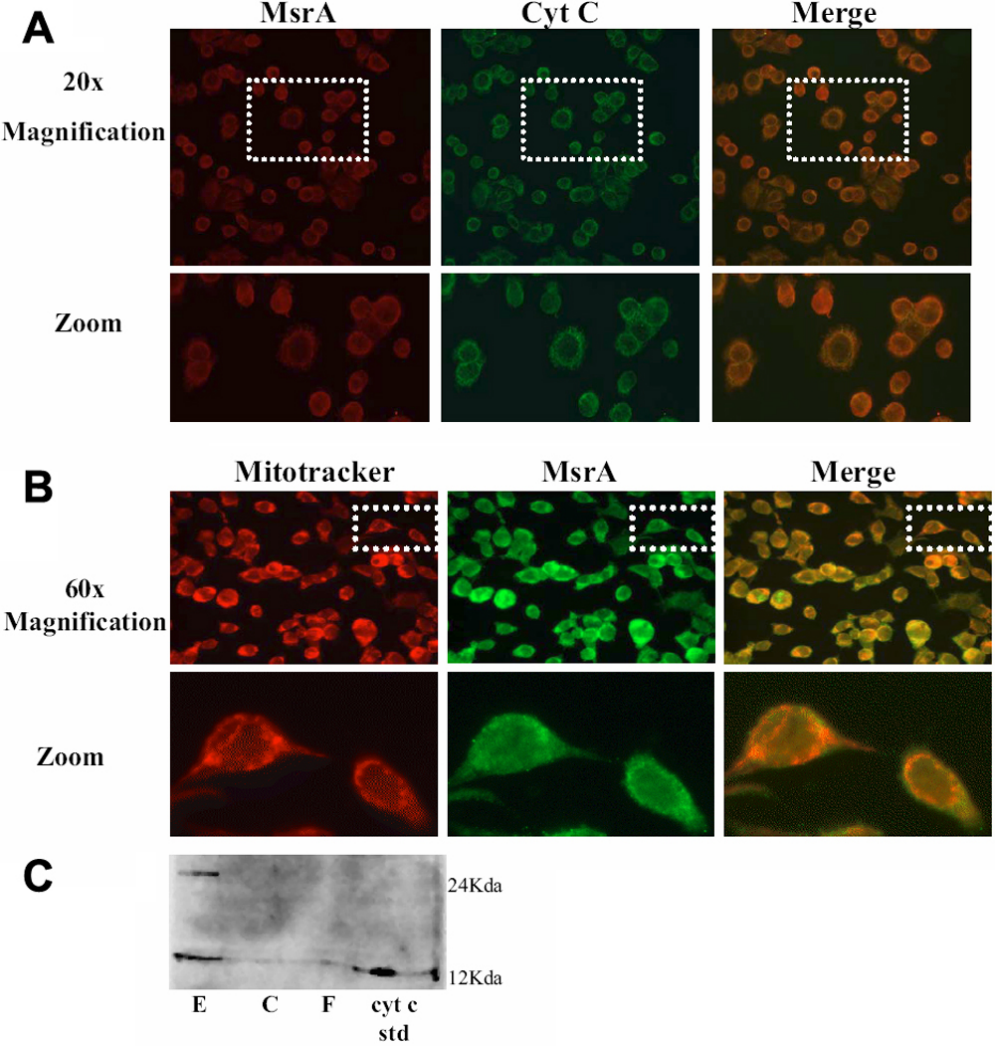
Co-localization of MsrA and cytochrome c in lens epithelial cells. **A**: Co-localization of MsrA (red) and cyt c (green) and (C) merging of the two images (orange) in human lens epithelial cells by immunofluoresence microscopy. The bottom panel shows a zoomed area of the indicated regions from the top panel. **B**: Co-localization of mitotracker mitochondrial marker (red) and MsrA (green) and (C) merging of the two images (orange) in human lens epithelial cells by immunofluoresence microscopy. The bottom panel shows a zoomed area of the indicated regions from the top panel. **C**: SDS-PAGE and immunoblotting of microdissected lens epithelium (E), cortex (C) and fiber (F) total protein extracts with a cyt c-specific antibody using 100 µg of protein.

Consistent with the co-immunoprecipitation studies, MsrA and cyt c co-localized in cultured human lens epithelial cells in vivo (Figure 3A) and MsrA was also localized to the mitochondria of human lens epithelial cells (Figure 3B). Cyt c was almost entirely confined to the lens epithelium in protein extracts isolated from microdissected human lens epithelium, cortex and nuclear regions (Figure 3C).

### Cytochrome c is aggregated and/or insoluble in the lenses of MsrA Knockout mice but not wild-type mice

Previous studies indicated that cyt c loses its ability to transport electrons in vitro upon methionine sulfoxide formation [17] and aggregates upon oxidation in vitro [26,27]. Given that MsrA and cyt c interact in lens cells, we next sought to determine the oxidation and aggregation states of cyt c in the knockout mice relative to the wild-type mice. Lens extracts were prepared from MsrA knockout and wild-type mice and cyt c examined for methionine oxidation and aggregation by CNBr cleavage and western blotting. CNBr cleaves proteins specifically at methionine and is inactive for cleavage of methionine sulfoxide. There are two methionines in mouse cyt c at positions 65 and 80. CNBr cleavage of methionines in mouse cyt c therefore results in the production of three peptides of molecular weights 7,146 kDa, 2,911 kDa, and 1,811 kDa. The 2,911 kDa peptide arises from cleavage at met 80 while the other two peptides result from cleavage at met 65 and met 80. CNBr cleavage of cyt c containing met 80 sulfoxide without containing met 65 sulfoxide would result in 7,277 kDa and 4,573 kDa peptides; while oxidation of met 65 in the absence of oxidation at met 80 would yield 8,939 kDa and 2,911 kDa peptides.

**Figure 4:**
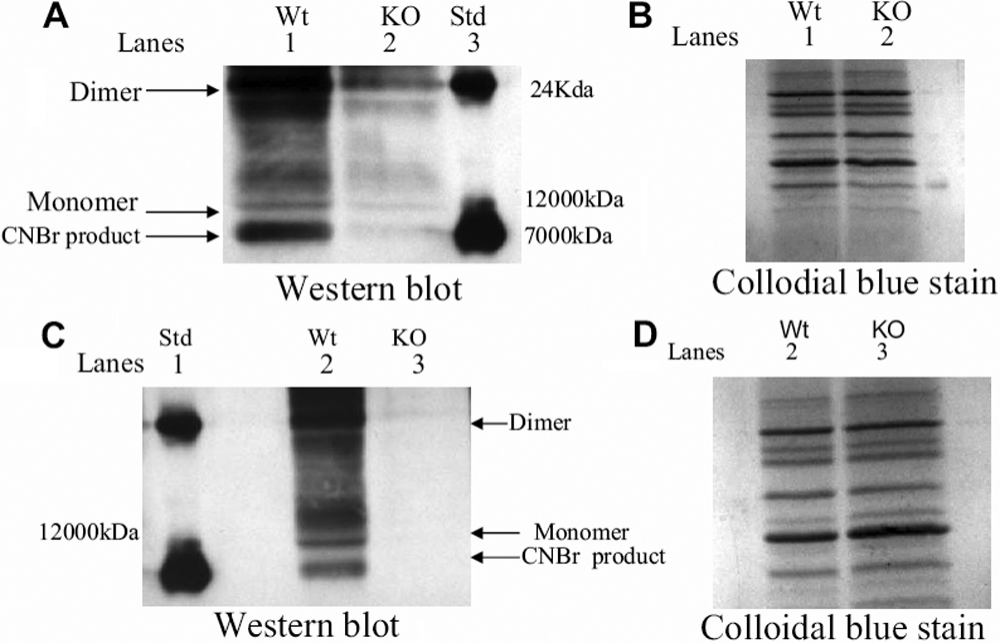
Cyt c in MsrA deficient mouse lenses shows evidence of oxidation and aggregation. **A**: SDS-PAGE and immunoblotting of cyt c (5 µg) following CNBr cleavage of total lens protein extract with a cyt c specific antibody. Lens protein extracted from wild-type and MsrA knockout mouse lenses with no HBO treatment. Lane 1 – wild type mouse lens, Lane 2 – MsrA knockout mouse lens, Lane 3 – cyt c standard. **B**: Colloidal blue staining of the SDS-PAGE gel shown as a control for protein loading. **C**: SDS-PAGE and immunoblotting of cyt c (5 µg) following CNBr cleavage of total lens protein extract with a cyt c specific antibody. Lens proteins extracted from wild-type and MsrA knockout mouse lenses following HBO treatment. Lane 1 – wild type mouse lens, Lane 2 – MsrA knockout mouse lens, Lane 3 – cyt c standard. **D**: Colloidal blue staining of the SDS-PAGE gel shown as a control for protein loading.

As expected, CNBr treatment of wild-type mouse lens protein resulted in the detection of an approximate 7,146 kDa band indicative of non-oxidized cyt c as well as uncut monomer and dimer forms of cyt c (see western blot Figure 4A, lane 1 and coomassie stain Figure 4B lane 1). The smaller predicted products of 2,911 and 1,811 required to determine specific cleavage at met 80 relative to met 65 could not be detected due to limitations in gel resolution. By contrast, almost no 7,146 kDa cleavage product was apparent in the MsrA knockout lens extracts as well as almost no monomer form of cyt c (western blot Figure 4A lane 2 and coomassie Figure 4B lane 2). The dimer form of cyt c was detected in small amounts (western blot Figure 4A, lane 2). These results suggest that cyt c is oxidized and aggregated in the lenses of the MsrA knockout mice consistent with previous studies demonstrating aggregation of cyt c upon oxidation in vitro [26,27]. These results appeared to be augmented by HBO treatment. Analysis of HBO treated wild-type lenses revealed similar monomer and CNBr cleaved forms (western blot Figure 4C lane 2 and coomassie stain Figure 4D lane 2). However, no monomer or dimer forms of cyt c could be detected in the HBO-treated MsrA knockout lenses (western blot Figure 4C lane 3 and coomassie stain Figure 4D lane 3).

### MsrA repairs oxidized cytochrome c in vitro resulting in cytochrome c de-aggregation

Since cyt c levels in the lens or lens cells were too low for accurate analysis, purified horse heart cyt c was used to investigate whether purified MsrA could actually repair methionine sulfoxide in cyt c. Aggregation of cyt c in the MsrA knockout lenses suggested that lens MsrA could repair cyt c methionine sulfoxide which could restore oxidized cyt c to a de-aggregated state. To test this hypothesis, purified cyt c (0.2mM; horse heart) was treated with increasing amounts of HOCl (2:1, 4:1, and 6:1 molar ratios) which specifically converts methionines in cyt c to methionine sulfoxide, and oxidized cyt c was incubated with or without MsrA with the addition of DTT as a reducing system. The resulting samples were treated with CNBr and run on tris-tricine gels. Cleavage of wild-type cyt c with CNBr should result in three peptides of molecular weights 7,146 kDa, 2,911 kDa, and 1,811 kDa. If both methionines in cyt c are oxidized no CNBr bands should be visualized. CNBr cleavage at met 80 should result in a 2,911 kDa band. CNBr cleavage of cyt c with oxidation at met 80 in the absence of oxidation at met 65 should result in 7,277 kDa and 4,573 kDa peptides; while oxidation of met 65 in the absence of oxidation at met 80 would yield 8,939 kDa and 2,911 kDa peptides.

**Figure 5:**
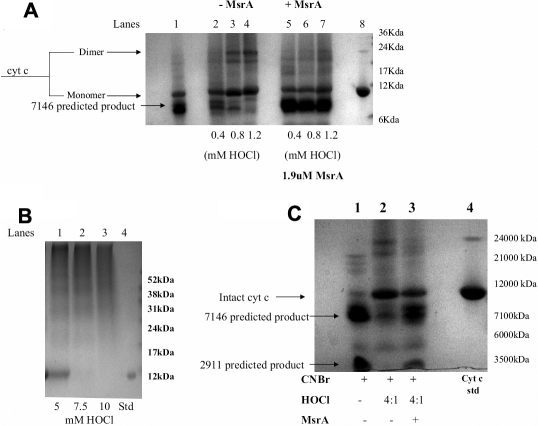
MsrA repairs oxidized methionines in cyt c and de-aggregates oxidized cyt c. **A**: Colloidal blue staining of a Tricine-SDS-PAGE gel following CNBr cleavage of oxidized cyt c (5 µg). Lane 1 untreated cyt c, lane 2 - 0.4 mM HOCl (2:1 molar ratio), lane 3 - 0.8 mM HOCl (4:1 molar ratio) and lane 4 - 1.2 mM HOCl (6:1 molar ratio). Lanes 5-7 are Cyt c (with the same ratios HOCl) repaired by 1.9 µM MsrA and 15 mM DTT. **B**: SDS-PAGE gel analysis of cyt c oxidized (5 µg) with high doses of HOCl using colloidal blue staining. Lanes 1 – 3, high doses of HOCl (5, 7.5 and 10 mM HOCl). Lane 4 contains the cyt c standard. **C**: Colloidal blue staining of a Tricine-SDS-PAGE gel of cyt c (5 µg) following CNBr cleavage. Lane 1 – Untreated cyt c cleaved with CNBr. Lane 2 – Oxidized cyt c (0.2 mM cyt c treated with 0.8 mM HOCl, 4:1 molar ratio) cleaved with CNBr. Lane 3 – Oxidized cyt c (291 µM) treated with MsrA (1.9 µM) and DTT (15 mM) for 2 h at 37 ºC and cleaved with CNBr.

Treatment of cyt c with increasing amounts of HOCl for 15 min resulted in a dose dependent loss of CNBr cleavage product at approximately 7,146 kDa signifying oxidation of both methionines in cyt c (Figure 5A, Lanes 2-4) upon treatment with HOCl. As expected, there was also a concentration dependent increase in aggregation of cyt c (Figure 5A, lanes 2-4). The formation of dimers and oligomers was observed with increasing concentrations of HOCl. The two smaller cleavage products of 2,911 kDa and 1,811 kDa were not visualized on this gel due to lack of resolution. Incubation of the oxidized aggregated cyt c (291 µM) with 1.9 µM MsrA for 2 h at 37 ºC resulted in repair of oxidized methionines in cyt c as evidenced by the return of the 7,146 kDa product in all three samples (Figure 5A, lanes 5-7). In addition, decreases in dimer and oligomer forms (Figure 5A, lanes 5-7) were also observed suggesting that the oxidation state of methionines is important for the aggregation state of cyt c. Increased levels of HOCl above 5 mM resulted in complete loss of monomer and dimer forms (Figure 5B, lanes 1-3).

Considerable evidence indicates that met 80, which co-ordinates the heme group in cyt c is essential for cyt c function in terms of electron transport [17] and may play a critical role in the ability of cyt c to initiate apoptosis [28,29]. Considering the potential importance of met 80, we wished to confirm met 80 oxidation upon HOCl treatment and the ability of MsrA to specifically repair cyt c met 80 sulfoxide. Incubation of 0.2 mM horse heart cyt c protein with 0.8 mM HOCl (4:1 molar ratio) resulted in oxidation of methionines at positions 65 and 80 as evidenced by the reduction in intensity of the 7,146 kDa band and non-detectable levels of the 2,911 kDa band following CNBr treatment (Figure 5C, Lane 2). Oxidized methionines in cyt c (291 µM), including met 80 were repaired by incubation with 1.9 µM MsrA for 2 h at 37 ºC with 15 mM DTT as evidenced by the increased intensity of the 7,146 kDa band and the reappearance of the 2,911 kDa band (Figure 5C, Lane 3) relative to the oxidized protein (Figure 5A Lane 2). In both cases, the 1,811 kDa band, consistent with oxidation of both met 80 and met 65 was not visible using these conditions. As a control, DTT alone had no effect on cyt c repair (data not shown). This data confirms specific oxidation of cyt c met80 and its repair by MsrA.

**Figure 6:**
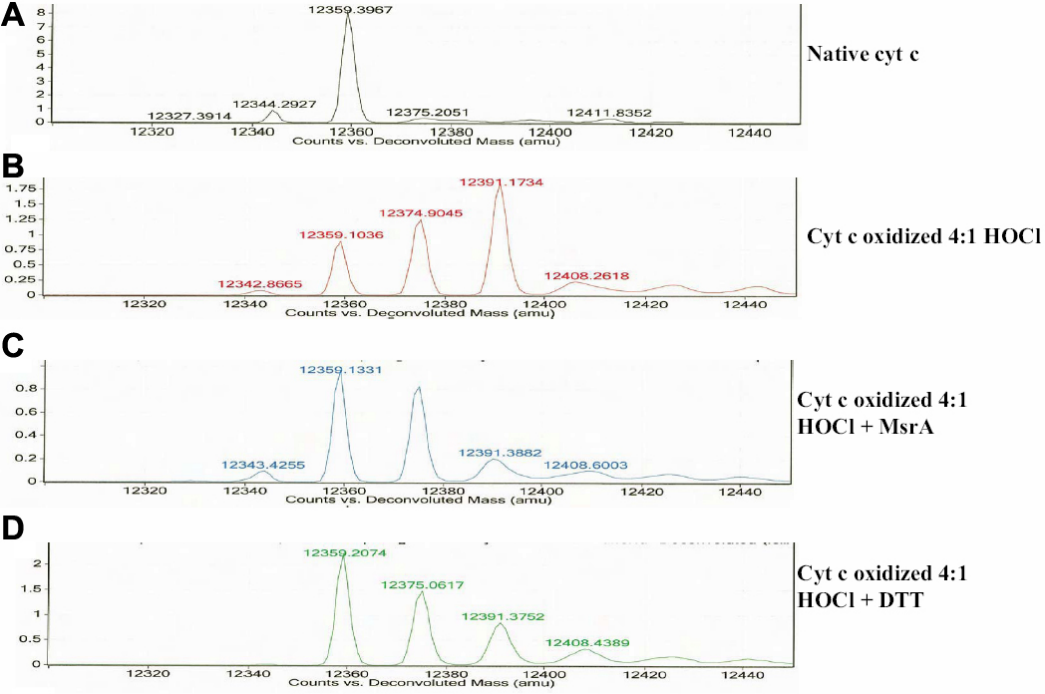
Deconvoluted ESI mass spectra of HOCl-oxidized cytochrome c. **A**: Native cytochrome c. **B**: Cyt c oxidized (4:1 Cyt c: HOCl for 15 min). **C**: Cyt c oxidized (4:1 Cyt c: HOCl for 15 min) and repaired by 1.9 μM MsrA (2 h at 37 ºC) and 15 mM DTT. **D**: Cyt c oxidized (4:1 Cyt c: HOCl for 15 min) and incubated in repair buffer containing 15 mM DTT only (2 h at 37 ºC).

As a secondary conformation, oxidation of cyt c was further analyzed by mass spectrometry (Figure 6A-D). The electrospray mass spectra of untreated cyt c contained a parent ion of average molecular weight – 12,359 (Figure 6A). The addition of one oxygen molecule yields a molecular weight of 12,374 while the addition of two oxygens results in a molecular weight of 12,391. Oxidation of cyt c with a 4:1 molar ratio of HOCl for 15 min led to both a one and a two oxygen addition (31% and 46% respectively of total cyt c). About 22% of cyt c remained in the native form (Figure 6B). Incubation of the oxidized cyt c (291 µM) with 1.9 µM MsrA for 2 h at 37 ºC led to a reduction to 10% of the two oxygen addition while the one oxygen addition went from 31% to 42% and the native increased to 48% (Figure 6C). Oxidized cyt c was incubated for 2 h at 37 ºC with repair buffer containing 15 mM DTT alone with no MsrA (Figure 6D). DTT alone had some reducing effect on the two oxygen addition as seen in Figure 5D with a reduction from 46% to 19% but not the one oxygen addition. Similar amounts of native protein were observed following DTT alone as seen with MsrA (49% and 48%). These results suggest that some reduction could be attributed to DTT despite DTT having no effect on the repair shown in Figure 5A and Figure 5C.

### MsrA restores cytochrome c function lost upon methionine oxidation

**Figure 7:**
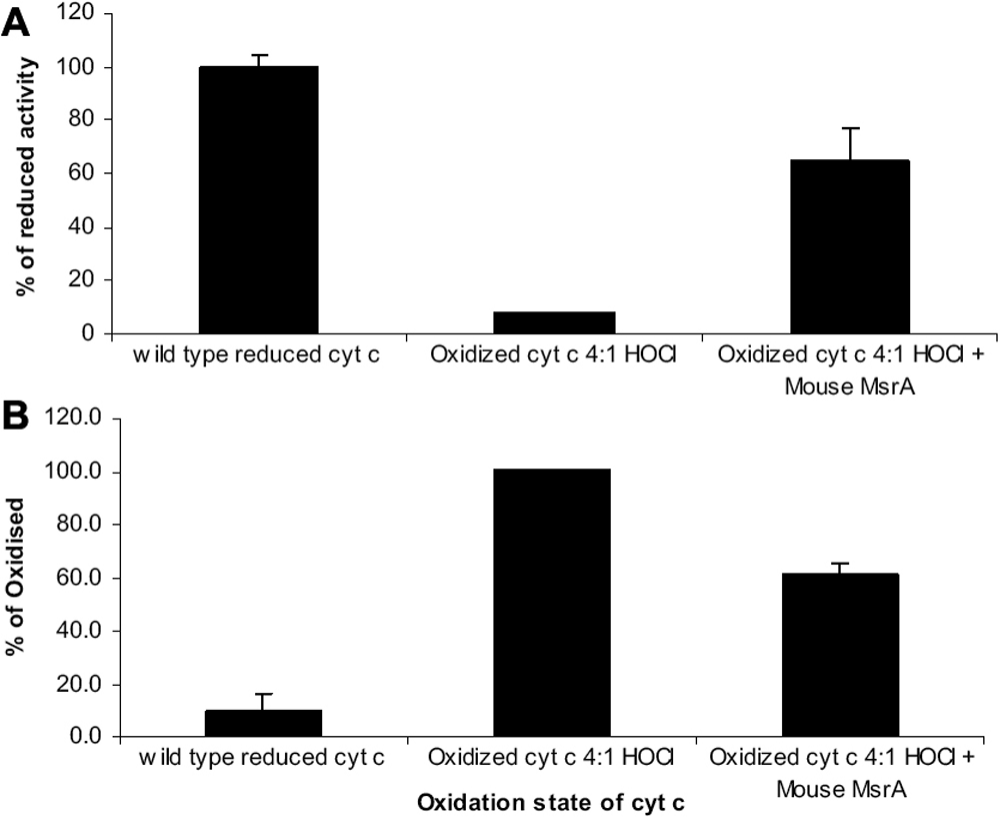
MsrA repairs the oxidase activity and decreases the peroxidase activity of cytochrome c. **A**: Representative graph of cytochrome c oxidase activity using oxidized and repaired cyt c. Cytochrome c oxidase activity using reduced wild type cyt c gives maximum activity in this assay. When oxidized cyt c (incubated for 15 min with a 4:1 molar ratio of HOCl) is used as a substrate for cytochrome c oxidase, the activity drops by 92% to 8.3% Treatment of the oxidized cyt c (291 µM) with MsrA (1.9 µM for 2 h at 37 ºC) and 15 mM DTT leads to a seven fold increase in cyt c oxidase activity to 66% or 58% repair above the oxidized. Activity shown here is as a percentage activity of the oxidized form of cyt c. **B**: Representative graph of cyt c mediated peroxidase activity. The wild type protein has 9.5% of the activity of the oxidized protein. Oxidized cyt c (incubated for 15 min with 4:1 molar ratio of HOCl) gives maximal peroxidase activity. Treatment of the oxidized cyt c (291 µM) with MsrA (1.9 µM for 2 h at 37 ºC) and 15 mM DTT leads to a 40% decrease in cyt c peroxidase activity. Activity is shown here as a percentage of the activity of the oxidized form of cyt c.

Previous work showed that cyt c could be preferentially oxidized on met 80 to met 80 sulfoxide in vitro through HOCl treatment [17] and the data presented here confirms oxidation of both methionines in cyt c and confirms met 80 oxidation. Chen et al. [17] also showed that met 80 oxidation inhibited the ability of cyt c to transfer electrons and increased the peroxidase activity. To determine if MsrA could repair cyt c inactivated through the formation of met 80 sulfoxide, we examined the oxidase and peroxidase activities of oxidized and repaired cyt c. Cyt c was oxidized and repaired exactly as described for Figure 4C. Oxidation of cyt c by HOCl resulted in a 92% loss of cytochrome c oxidase activity (Figure 7A) when compared to the wild type reduced cyt c. Treatment of the oxidized cyt c with MsrA restored about 58% of the activity of reduced cytochrome c oxidase. This is in excess of the 50% repair of cyt c expected on the basis of the S-epimer specificity of the enzyme and the predicted 50% S-epimer formation of cyt c met 80 sulfoxide resulting from HOCl chemical oxidation. As a control, incubation with DTT alone had no effect on cytochrome c oxidase activity using oxidized cyt c (data not shown). Conversion of cyt c to cyt c met 80 sulfoxide is known to increase peroxidase activity of cyt c [17]. Oxidation of cyt c resulted in increased peroxidase activity compared to the wild type proteins – oxidized is designated as 100% here, the wild type cyt c has 9.5% activity in comparison (Figure 7B). Consistently, treatment of oxidized cyt c with MsrA resulted in a 39% reduction in cyt c peroxidase activity in excess of the effect of DTT alone (Figure 7B).

## Discussion

Lens cataract is an oxidative stress disease associated with protein methionine oxidation [2,3] and MsrA which repairs oxidized methionines has been shown to be important for lens cell viability [10] and interestingly, lens cell mitochondrial function [11]. MsrA is also a major regulator of lifespan in mice [6] and is linked to a multitude of diseases [30-34] including the aging process itself [35,36].

Loss of MsrA activity could account for increased levels of PMSO found in cataract relative to clear lenses. We hypothesized that cataract could be a cumulative event caused at least in part by failure of MsrA to repair damaged proteins. Since loss of mitochondrial function was detected in lens cells upon silencing of the MsrA gene [11], we further hypothesized that key mitochondrial proteins could be targets for MsrA activity.

One key component for the maintenance of the lens is the lens epithelium, which contains the highest levels of enzymes and transport systems in the lens [37], it is essential for the growth, differentiation and homeostasis of the entire lens [38] and contains high levels of mitochondria. It has been shown that damage to the lens epithelium and its enzyme systems, can result in cataract formation [39-42]. Oxidative damage to the lens epithelium is believed to be an initiating factor in cataract formation [40], is associated with extensive DNA damage, damage to membrane pump systems (Ca ATPase, Na/K ATPase) and loss of reduced GSH. Collectively all of these could cause damage to the underlying fiber cells and result in cataract formation [40,42]. In addition, damage to the mitochondria of lens epithelial cells may result in apoptosis which has been associated with cataractogenesis [23-25]. Many of these systems rely on ATP and reducing systems provided through mitochondrial activity.

In the present report, we examined the lenses of HBO-treated and aged-matched MsrA knockout mice relative to wild-type mice and we detected increased light scattering in the HBO-treated knockout mice. We do not know if further aging of MsrA knockout mice would result in cataract and therefore cannot on the basis of these findings determine whether HBO treatment accelerates normal aging or stresses the lens in excess of normal aging. However, it is believed that HBO treatment results in changes similar to those found in aged human lenses [12]. Nevertheless, these findings do suggest that MsrA knockout lenses exhibit an increased sensitivity towards exogenous oxidative stress (Figure 1B-E). Light scattering in these lenses appeared to be localized to the lens nucleus suggesting that light scattering is likely the result of oxidized and aggregated crystallins. The lens epithelium is the first site of oxygen exposure and a large body of evidence indicates that epithelial damage is an initiator of cataract. Given that MsrA protein is mainly localized to the epithelium [10] and is required for the maintenance of lens cell mitochondrial function and epithelial resistance to oxidative stress we focused on the mitochondria of the lens epithelium as a likely site for the action of MsrA in preventing damage to and/or repairing lens proteins that could be involved in increased light scattering in HBO treated MsrA knockout mice.

Co-immunoprecipitation studies with lens epithelial cell extracts and antibodies specific for the five major mitochondrial complexes, cyt c and MsrA revealed that MsrA interacted with all of the above mitochondrial components except for complex IV where a band size inconsistent with complex IV was detected (Figure 2A). Since the mitochondrial complexes consist of many proteins and given the importance of cyt c as both an electron carrier in the respiratory chain and a modulator of apoptosis, we focused on cyt c as a possible target for MsrA. Oxidation of cyt c in the MsrA knockout lens and loss of its activity could damage the lens epithelium through loss of electron transport, loss of cationic exchange and apoptotic induction, all of which could ultimately contribute to increased light scattering and cataract formation.

Consistent with a potential role for MsrA repair of cyt c in the lens, analysis of the MsrA knockout lenses revealed increased oxidation and aggregation of cyt c that was not found in wild-type lenses (Figure 4A) this was exacerbated following HBO treatment where cyt c was no longer detected in the soluble protein (Figure 4C). Indeed, little soluble cyt c could be detected at all in these lenses consistent with previous studies showing that oxidized cyt c forms higher molecular weight aggregates and that oxidized cyt c degrades in vitro [17,26,27]. To further explore whether MsrA can repair oxidized cyt c, the ability of MsrA to repair cyt c was evaluated using purified cyt c and MsrA. CNBr treatment, which specifically cuts reduced but not oxidized methionine in cyt c revealed specific methionine oxidation that could be repaired by MsrA treatment (Figure 4A). Consistent with the aggregation observed in the knockout lenses, high levels of oxidant (HOCl) resulted in increased aggregation and degradation of cyt c in vitro (Figure 5B). Further analysis of the oxidized cyt c protein by CNBr treatment revealed two sites of methionine oxidation at met 80 and met 65 (Figure 5C). Oxidation levels were further confirmed by mass spec analysis (Figure 6A-D). Consistent with these oxidations being important for cyt c function, methionine oxidation of cyt c reduced its activity as an electron carrier by 92% (Figure 7A) and increased cyt c peroxidase activity tenfold (Figure 7B) as previously reported [17]. MsrA treatment of oxidized cyt c restored cyt c oxidase activity to 58% of the wild-type reduced protein and reduced the oxidized cyt c peroxidase activity by 39% (Figure 7A,B). Repair by MsrA of cyt c oxidase activity was in excess of the expected 50% based on S-epimer specificity and the expected random 50-50 distribution of S- and R- epimers in the oxidized cyt c while for cyt c peroxidase activity repair in terms of percent activity was slightly lower than expected. In effect, the R- and S-epimer distribution is unlikely to be strictly 50-50 and the amount of repair found here reflects that situation. This data indicates that MsrA efficiently repairs oxidized methionines at low enzyme concentrations, here the ratio of oxidized protein to enzyme was 153:1.

With this HBO model the levels of oxygen are likely to be higher in the epithelial and periphery than in the center of the lens through diffusion of oxygen through the cornea as shown by Shui et al. [43], potentially targeting the cells of the lens epithelium which as stated above can be the initiating site for cataractogenesis. Damage following HBO treatment of guinea pigs or rabbits involves disulfide formation, membrane damage and loss of cytoskeletal proteins as well as decreased GSH and ascorbate levels in the nucleus and cortex and β- and γ-crystallin aggregation, all ultimately leading to nuclear cataract [12,44-46]. HLE B3 cells treated with HBO showed a 30% decrease in ATP levels after 3 h of treatment consistent with loss of mitochondrial function. Indeed, transmission electron microscopy analysis of those cells at 16 h indicated a decrease in the total number of mitochondria [14]. Haung et al. [13] cultured HLE B3 cells in hyperoxia conditions and showed increased mitochondrial ROS (43%), decreased mitochondrial membrane potential and loss of cardiolipin, a key player in the regulation of the mitochondrial apoptosis [47]. All of these studies point to loss of mitochondrial function as an important characteristic of lens oxidation and suggest that MsrA repair of oxidized lens mitochondrial proteins is a key factor in lens maintenance.

In addition to serving as targets for MsrA, methionine residues are thought to act as antioxidants by directly scavenging ROS, lack of MsrA to reverse this process may increase not only PMSO but endogenous ROS that can no longer be scavenged. This may explain the oxidation and aggregation of cyt c in vivo even without HBO treatment. These results correlate well with the findings of Marchetti et al. [11] where loss of cell viability, decreased mitochondrial membrane potential and increased ROS levels were found in HLE B3 cells silenced for MsrA, even in the absence of oxidative stress. Since HBO treatment results in changes similar to aged human lens [12] the model used here could reflect events in aged human lenses in the presence or absence of MsrA, highlighting the importance of this enzyme as both a repair system and antioxidant scavenger. Interestingly, Shur-Perek and Avi-Dor [48] showed that the dimer form of cyt c, found here in MsrA knockout mice, was relatively ineffective as an electron carrier in the respiratory system. Upon HBO treatment of MsrA knockout mice the cyt c protein appears to be completely degraded or aggregated to an insoluble form which was also observed in Figure 4B with high concentrations of HOCl (5-10 mM) and observed by Chen et al. [17] when ratios of HOCl:cyt c above 8:1 were used. It could be that in the absence of MsrA activity, the mitochondrial system is overwhelmed with ROS, damaging not just cyt c but other key mitochondrial proteins and eventually causing lens epithelial cell apoptosis and ultimately cataract.

It has been proposed that the main function of mitochondria in the lens is to keep oxygen tension low [49]. Mitochondria consume about 90% of the oxygen entering the cell and when uncoupled, the lens sharp focus is lost [50]. Without MsrA functioning as both an antioxidant and repair system, oxygen consumption may decrease, leading to mitochondrial dysfunction, increased ROS production and ultimately apoptosis. In bovine lenses at least 30% of the energy used in the form of ATP is derived from mitochondrial respiration [51]. Loss of this energy is likely destructive to the lens.

In this report, we show that one key protein likely to be oxidized under these conditions is cyt c. In vitro methionine oxidation in cyt c led to decreased electron transport ability and increased peroxidase activity. Decreased electron transport ability leads to mitochondrial dysfunction which is seen in a number of studies using hyperoxia and cultured lens epithelial cells. Previous studies have shown that only the oxidized form of cyt c has the ability to initiate apoptosis [52].

**Figure 8:**
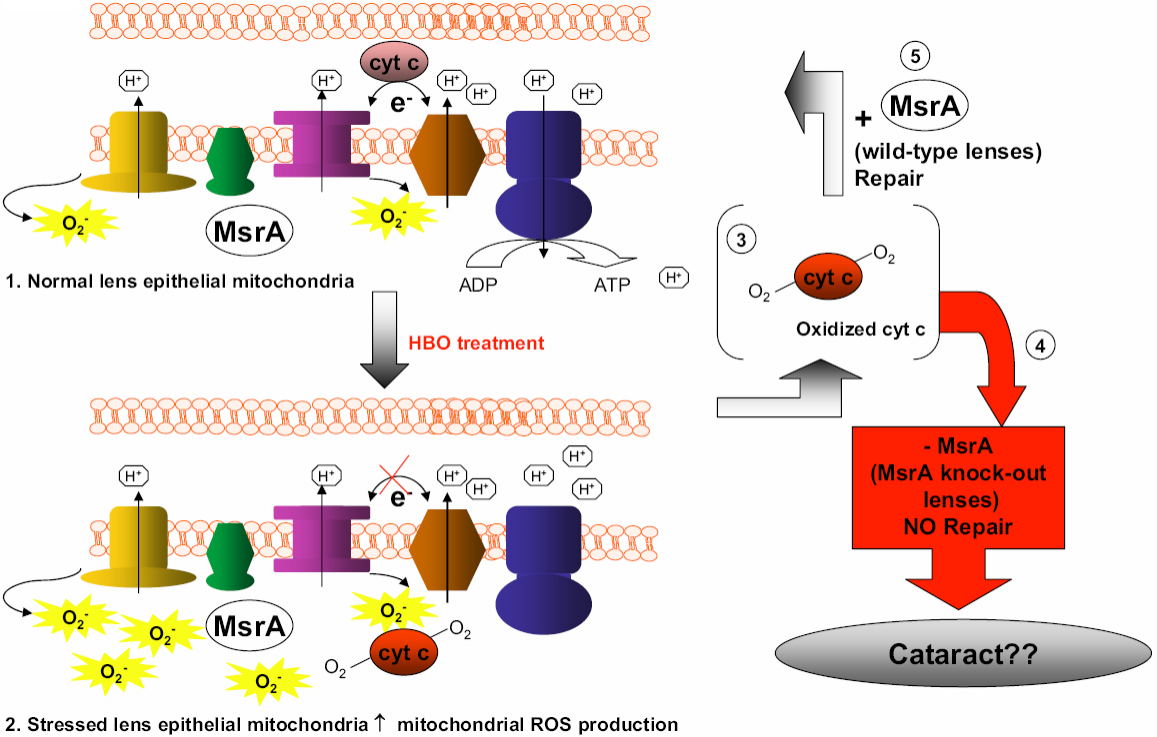
Model of MsrA repair of cyt c to maintain lens function and potential consequences on lens function of un-repaired oxidized cytochrome c. During normal respiration in the lens cyt c functions to transfer electrons from complex III to complex IV of the electron transport chain (ETC) (1), superoxide is a by product of the ETC and mainly comes from complexes I and III. On exposure to HBO treatment an increase in ROS can cause mitochondrial dysfunction, reduced or no electron transport and increased ROS production (2). Cyt c may become oxidized on its methionine residues as a consequence of oxidative stress (3). Cyt c may be then released by the mitochondria to initiate the internal pathway of apoptosis (4) or be repaired by MsrA (5) to restore normal function. Lack of MsrA repair of cyt c could results in loss of a multitude of lens functions and ultimately cataract.

In summary, the present report provides evidence that lack of MsrA results in light scattering in mice exposed to HBO treatment. Based on the mitochondrial location of both MsrA and cyt c and based on the importance of the lens epithelium for lens homeostasis, we chose to examine cyt c as a potential substrate for MsrA in the lens. A model of these findings is shown in Figure 8. We detected methionine oxidation of cyt c in vivo that was repairable by MsrA in vitro. MsrA action restored the function of cyt c in vitro suggesting that in the absence of MsrA oxidative stress could lead to loss of electron transport, increased production of ROS, loss of lens transport, apoptosis and ultimately cataract. Cataract likely occurs through the over oxidation, inactivation and ultimately aggregation of multiple key lens proteins (Figure 8). Clearly cyt c is not the only lens protein acted upon by MsrA and its activity is likely one of the many important lens functions damaged through methionine oxidation. Nevertheless, identification of cyt c as oxidized in MsrA knockout mice lenses and establishing MsrA that can restore the normal function of cyt c is an important first step in defining the mechanisms underlying the role of MsrA in lens mitochondrial maintenance, repair and ultimately cataract formation.
